# Molecular Dynamics Simulations Suggest a Non-Doublet Decoding Model of −1 Frameshifting by tRNA^Ser3^

**DOI:** 10.3390/biom9110745

**Published:** 2019-11-18

**Authors:** Thomas Caulfield, Matt Coban, Alex Tek, Samuel Coulbourn Flores

**Affiliations:** 1 Department of Neuroscience, Mayo Clinic, Jacksonville, FL 32224, USA; 2 Department of Cancer Biology, Mayo Clinic, Jacksonville, FL 32224, USA; Coban.Mathew@mayo.edu; 3 Department of Cell and Molecular Biology, Uppsala University, 751 24 Uppsala, Sweden; alex.tek.se@gmail.com; 4 Department of Biochemistry and Biophysics, Stockholm University, 10691 Stockholm, Sweden

**Keywords:** ribosome, −1 frameshifting, S13, doublet decoding

## Abstract

In-frame decoding in the ribosome occurs through canonical or wobble Watson–Crick pairing of three mRNA codon bases (a triplet) with a triplet of anticodon bases in tRNA. Departures from the triplet–triplet interaction can result in frameshifting, meaning downstream mRNA codons are then read in a different register. There are many mechanisms to induce frameshifting, and most are insufficiently understood. One previously proposed mechanism is doublet decoding, in which only codon bases 1 and 2 are read by anticodon bases 34 and 35, which would lead to −1 frameshifting. In *E. coli*, tRNA^Ser3^_GCU_ can induce −1 frameshifting at alanine (GCA) codons. The logic of the doublet decoding model is that the Ala codon’s GC could pair with the tRNA^Ser3′^s GC, leaving the third anticodon residue U36 making no interactions with mRNA. Under that model, a U36C mutation would still induce −1 frameshifting, but experiments refute this. We perform all-atom simulations of wild-type tRNA^Ser3^, as well as a U36C mutant. Our simulations revealed a hydrogen bond between U36 of the anticodon and G1 of the codon. The U36C mutant cannot make this interaction, as it lacks the hydrogen-bond-donating H3. The simulation thus suggests a novel, non-doublet decoding mechanism for −1 frameshifting by tRNA^Ser3^ at Ala codons.

## 1. Introduction

In the ribosome, messenger RNA (mRNA) is most often decoded by reading in three-base (codon) steps, by cognate transfer RNAs (tRNAs), until a stop codon is encountered. However, frameshifting, misincorporation, premature termination, and stop-codon read-through [1] also occur. This can be part of natural regulatory processes, and are heavily used by viruses [2]; they can also play a role in tumorigenesis [3]. Lastly, they occur at low frequency, apparently as errors. Structures of the ribosome showing cognate and near-cognate interactions [4,5] have given us considerable understanding of decoding. However, −1 frameshifting remains difficult to characterize by experimental structural biology.

Ribosomes are large complexes, consisting of ribosomal RNA (rRNA) and a multitude of proteins. Prokaryotic (70S) ribosomes are divided into small subunit (30S) and large subunit (50S); the overall composition is about 2/3 rRNA and 1/3 protein, by mass. The ribosomes effect translation by pairing messenger RNA (mRNA) codons with transfer RNA (tRNA) anticodons, which carry amino acids that the ribosome then ligates to form a polypeptide chain. Codon–anticodon pairing occurs with high fidelity, even in prokaryotes, which lack some of the sophisticated error checking machinery possessed by eukaryotes; the average frequency for misreading in *E. coli* is 2.3 × 10^−6^ per codon [6].

Frameshifts can be caused by mutations in mRNA or tRNA, or by disruptions of codon–anticodon interactions through other mechanisms [2], which alter the triplet base reading frame. The three-base reading frame is most readily perturbed by single-base slippage in the backwards (5′) direction, called −1, or in the forward (3′) direction, called +1 frameshifting. It is possible to shift by more than a single base, e.g., −2 [7,8] or +5 [9], but these are rarer events with fewer examples. Frameshifting can be extremely deleterious when it occurs as an error, such as with Huntington’s disease [10,11,12] or Machado–Joseph disease [13,14]. In these cases, disease is caused by the neurotoxic accumulation of products, such as polyalanine, which are resultant from frameshifts in unstable codon reiteration sequences. Random occurrence of frameshifts has a very low frequency, estimated on the order of 1 in 10^5^ codons read [15].

Frameshifting also occurs natively. Programmed frameshifting is a well-documented phenomena [16]; it has been found in viruses [17,18], bacteria [19], yeast [2,20], and humans [21,22]. This is thought to be a genetic regulation and/or multiplexing method—to increase the number of proteins that can be encoded in a limited amount of genetic material [2], which is likely why examples are abundant for viruses and prokaryotes.

Mitochondrial ribosomes, or mitoribosomes, have a uniquely different composition from cytoplasmic ribosomes where the composition of RNA to protein is switched from 2:1 to 1:2, which allows for the nucleus to control mitochondrial protein synthesis [23]. Despite this drastic change in morphology of the ribosome, there is still evidence for frameshifting within the mitoribosome [24]. However, the internal regions of both ribosomes are highly conserved [23].

The anticodon stem-loop (ASL) is the portion of the tRNA that contains the anticodon and adjacent bases that maintain the conformation of the loop, a total of seven bases (32 to 38) in the canonical system. Most structural studies investigate canonical codon–anticodon interactions. However, there are an increasing number of structural analyses that are dedicated to non-standard codon–anticodon interactions, mostly demonstrating +1 frameshifting mechanisms (−1 frameshifting structures appear harder to obtain, possibly for stability reasons). An original study shows the 30S subunit in complex with +1 frameshift-inducing tRNAs with their cognate mRNA, evincing the ability of the ribosomal A site to harbor a quadruplet codon–anticodon interaction [25]. Another study shows an insertion between nucleotides 31 and 32 in ASL^SufJ^ causes distortion of the tRNA, which results in +1 frameshifting [26]. A similar tRNA frameshift suppressor is ASL^SufA6^ [27,28,29]. Thus, the quadruplet decoding mechanism for +1 frameshifting is well characterized.

**Figure 1:**
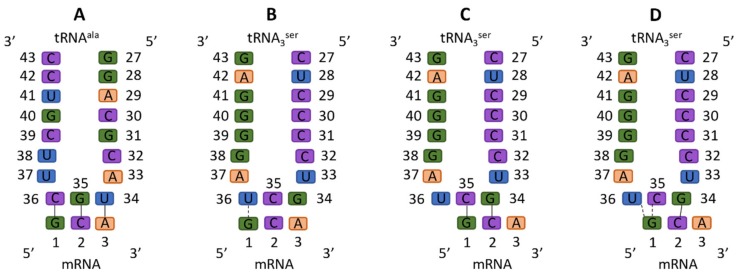
Standard and non-standard codon–anticodon interactions in the A site. Solid connectors indicate a Watson–Crick (WC) base pair, whereas dashed connectors indicate a non-standard interaction. (**A**) Standard cognate binding, in which all three WC base pairs are formed. (**B**) Binding of the frameshift-inducing tRNA in a standard way, where only one wobble base pair can be formed. (**C**) Doublet decoding model: The frameshift-inducing tRNAs bind to the frameshifting sites with two WC base pairs formed. Notably, as tRNA base 36 is not involved, under that model the U36C mutation should have no effect on tRNA^Ser3^ frameshifting. (**D**) Straddling interactions of mRNA G1 with tRNA U36 and C35, as observed in our simulations and described in the text.

In contrast, −1 frameshifting is not nearly as well understood. The first study suggesting a −1 reading frame was in 1979, showing that synthesis of a 66kDa MS2 synthetase, rather than the normal 62kDa form, was induced by tRNA^Ser^ or tRNA^Thr^, suggesting a reading frame change [30]. Furthermore, they used clever biochemistry to demonstrate the protein sequence was parsimoniously consistent with a −1 frameshift [30]. In Figure 1, we exhibit both standard and nonstandard Ser anticodon interactions. The mechanism for −1 frameshifting has been largely proposed to be nonstandard interactions of the frameshift-inducing tRNA at the frameshift site, namely tRNA bases 34 and 35 forming Watson–Crick pairs with mRNA bases 2 and 1, respectively; this is the doublet decoding model.

Because tRNA base 36 is not involved in any Watson–Crick pairing, its identity is not important in the doublet decoding model, ergo any mutation of U36 should not affect tRNA^Ser3^ frameshifting. However, it was observed decades ago that other tRNAs, which can form doublet G-C base pairs that do not have U at position 36, are incapable of inducing frameshifting [31]. As such, it was posited that U36 was a critical component for frameshifting, though the mechanism was suggested as allowing the anticodon to contort into a conformation, which allows for doublet decoding to occur [31]. Our modeling of the wild type U36 and the sterically conservative U36C mutation suggests an alternative non-doublet model to explain this phenomenon. In this study, we set out to computationally test the doublet decoding hypothesis, using both wild-type (U36) and U36C mutant tRNA^Ser^, and to characterize the mechanism of −1 frameshifting by this tRNA on Ala codons. We used MacroMoleculeBuilder (MMB) [32], to model sterically allowed frameshift-inducing tRNA conformations within the ribosome, which were subjected to Maxwell’s demon molecular dynamics (MdMD) simulations to reach energetically feasible states that could explain the biochemical data.

## 2. Materials and Methods

### 2.1. MMB Modeling

**Figure 2:**
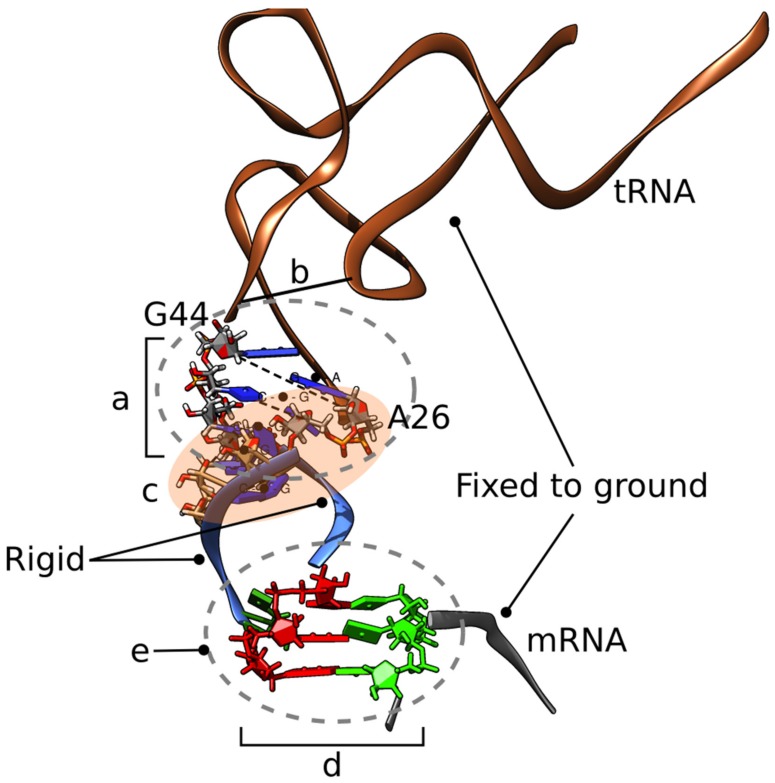
Structure, flexibility, and forces used to create the initial doublet-decoding models. (**a**) The tRNA is flexible around the hinge formed by residues 26, 27 and 43, 44. Physics is enabled for these residues. (**b**) Residue Y44 is constrained to residue 10. (**c**) Residues 26–30 and 44–40 form a nucleic acid duplex. Residues 25–28 have imposed helical stacking forces. (**d**) Anticodon residues 34–36 and codon residues 1 –3 are flexible. Base pairing is detailed below for each model. (**e**) Physics enabled for all residues within 1 nm of 35. With this multiscale setup, the Watson–Crick codon–anticodon base pairing forces (d) can simply be shifted from the initial triplet-triplet shown, to a doublet–doublet interaction to generate a clash-free doublet model. Not on figure: Springs enforce interactions with monitor residues 1492, 1493, and 530. It is essential to maintain the mRNA and anticodon stem-loop (ASL) in position. Protein S13 is flexible from residue 122 to 126 (C-ter) to remove clashes with the tRNA and position the residue 126 sidechain, which is absent from the crystal structure. A spring of 0.3 nm equilibrium length mimics the interaction of S13 residue R125 with a putative MG ion coordinated with 23S residue A1913 phosphate group. Collision-detecting spheres are placed on all heavy atoms within 1 nm of S13 residue 125 to prevent steric clashes.

**Figure 3:**
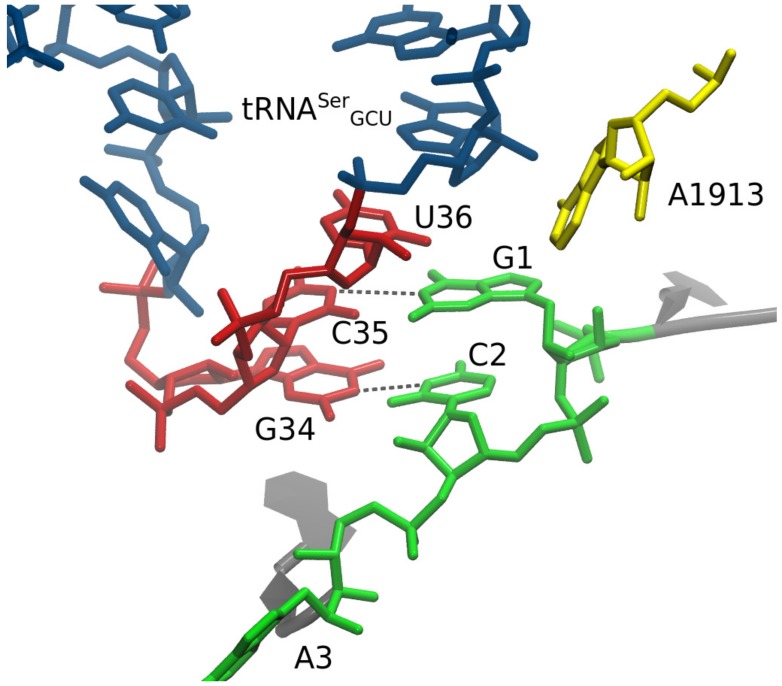
Construction of initial model under the doublet decoding hypothesis, using MacroMoleculeBuilder (MMB). Alanine GCA codon with tRNA^3Ser^ GCU. U36 is not engaged in any base pairing. Base pairs: (tRNA) G34 –C2 (mRNA); (tRNA) C35 –G1 (mRNA). Residues Y 35-36-37 have imposed helical stacking. The subsequent molecular dynamics (MD) simulation quickly shifted G1 to interact with U36; see main text.

**Figure 4:**
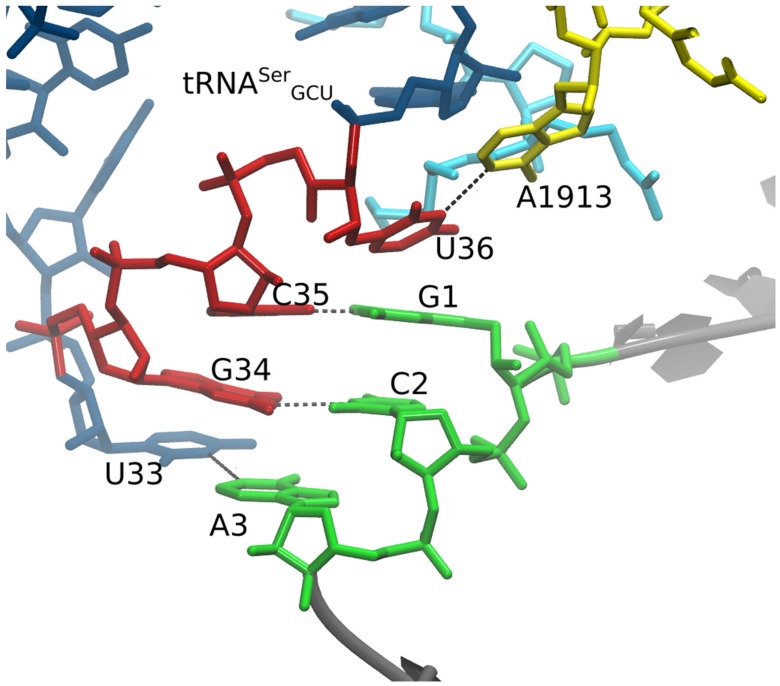
Grapple hypothesis. In this model, we enforced a U36 – A1913 base pair and U33 “grapple” Watson–Crick to Watson–Crick base pair with A3. The U33/A3 interaction was not maintained in the MD simulation. A1913 is a 23S subunit residue, whereas our experiments included only the small subunit. We therefore rejected the grapple hypothesis, as well as the U36-A1913 base-pairing hypothesis.

We created several MMB 3D structural models representing initial hypotheses. One is the cognate GCA/tRNA^Ala^ codon/anti–codon interaction, serving as control. The remainder show variants of the frameshifted tRNA^Ser3^, following variants of the doublet decoding model, also using MMB (Figure 2). The first is the wild-type tRNA^Ser3^ at a GCA codon (also referred to as U36), strictly following the doublet decoding model (Figure 3). The second is the mutant tRNA^Ser3^_U36C_, which aside from the U36C substitution is structurally identical to U36, and thus also following doublet decoding. We also made a U33 “grapple” structure, in which tRNA residue U33 (which is not in contact with mRNA in the cognate interaction) base pairs with mRNA residue A3. A variant was made in which U36 interacts with 23S residue A1913 (Figure 4). Lastly, we created a U36C-G1A double mutant, which, aside from the two substitutions, was structurally identical to the U36 model.

We built the models with MMB 2.15 using a multiscale approach. MMB allows users to adjust degrees of freedom bond by bond; for instance, to flexibilize only regions of interest. It also applies atomic forces, including simple collision-detecting spheres [33], or an atomistic molecular dynamics (MD) force-field (Amber99), restricted to user-specified regions. MMB can enforce all catalogued [34] base-pairing interactions using translational and rotational springs [33,35], and also apply other restraints and constraints.

The starting structure was based on a structure of the *Thermus thermophilus* 70S ribosome with complete A- and P-site tRNA in the pre-peptidyl transfer state [36], PDB entries 2WDK (30S), and 2WDL (50S). MMB simulations were run on a reduced model created by extracting all residues with atoms within 30 Å of the A-site tRNA, allowing quicker iterations for testing different structural hypotheses. This reduced model was thus composed of the complete A-site tRNA, mRNA, disparate parts of ribosomal RNA from both subunits, and the C-terminal part of ribosomal protein S13.

MMB routines were used to mutate the codon and ASL residues of the tRNA (tRNA^Phe^ in 2WDK) [36] to desired sequences. Upon mutation, MMB keeps the conformation the same for like-named atoms, and uses default bond lengths, angles, and dihedrals for any atoms in the mutant that are not present in the wild type. Very short equilibrations were used to remove inevitable clashes by making the concerned residues flexible and using collision-detecting spheres for surrounding atoms [35]. Collision-detecting spheres impose repulsive forces when an internuclear distance is lower than a predefined threshold; these are more economical than Lennard-Jones forces.

The approach of basing the tRNA^Ser3^ GCU model on tRNA^Phe^, with no post-transcriptional modifications, was motivated as follows. Position C32 natively has a 2-thio modification, but its absence did not affect translational efficiency [37]. Position A37 of tRNA^Ser3^ has a N6-threonylcarbamoyl modification [38]. However, this is expected to affect *stability* [29] of the *anticodon* [38], whereas we observed a conformational change in the *codon*.

In a second step, we defined the flexible parts and the interaction zones. The tRNA was flexibilized around the hinge formed by residues 26–27 and 43–44 (opening of the ASL), for which residues a force-field zone was established. A nucleic acid duplex structure was enforced for residues 26–30 and 40–44, residues 25–28 had imposed helical stacking, and residue 44 was welded to residue 10; fixed in space to avoid drifting. Anticodon residues 34–36 and mRNA codon residues 19–21 were flexible. Base pairing was enforced according to the scenario simulated. Amber99 atomic forces were applied to residues within 10 Å of anti-codon residue 35.

Springs of 2.5 Å equilibrium length enforced interactions with monitor residues 530, 1492, and 1493 of the small sub-unit to maintain the mRNA and ASL in position.

Protein S13 was flexible from residue 122 to 126 (C-ter) to remove clashes with the tRNA and position the residue 126 sidechain, which was absent from the crystal structure. A spring of 3 Å equilibrium length mimicked the interaction of S13 residue 125 with a supposed Mg2+ ion coordinated with the large sub-unit 1913 phosphate group.

Contact spheres were used for all atoms within 10 Å of S13 residue 125; everything else was rigid. In a third step, we equilibrated each system, and could then compare the different resulting structures to assess their structural feasibility. The multiscale model is shown in Figure 2.

We selected the U36 and U36C structures for more accurate MD simulations. Based on the results, we then also did an MD simulation of U36C-G1A.The whole ribosome was reconstituted by structurally aligning the reduced models onto the original full structure. The parts of our reduced model that had been flexibilized then replaced the corresponding atom in the original structure. Resulting clashes were then detected using UCSF Chimera [39] and removed with MMB as described above.

### 2.2. Molecular Dynamics (MD) Simulations

#### 2.2.1. Mutant Ribosome Structure Preparation

Following the MMB modeling of the three ribosome variants, double mutant U36C-G1A, U36 stack, and U36C, we imported into the structural modeling package to add hydrogens, protonate, and assign charges, which resulted in 247,776 atoms, 252,821 atoms, and 252,821 atoms, respectively. The refinement modeling was built using Schrodinger Maestro [40,41] and visual molecular dynamics (VMD) [42]. Fully solvated ribosomes were generated, with 2.9 to 3.1 × 10^6^ atoms. Calculations were completed on the Rochester Beowulf cluster and the North Supercomputing resource in Sweden.

Ribosomal proteins from the large and small subunit were inspected and validated using Procheck and What-If Krieger, Koraimann [43,44]. The side chains and rotamers were examined using refinement protocols and were verified [44]. The final model was subjected to energy optimization with Polak–Ribiere conjugate gradient (PRCG) with an R-dependent dielectric for 100,000 steps with relaxing restraints. Each model was exported to Maestro (MAE) and VMD (PDB) compatible formats (Supplementary Material). Model manipulation was done with Maestro [40,41] or VMD [42].

#### 2.2.2. Molecular Dynamics Simulation Protocol

The primary purpose of MD for this study was to probe the detailed codon–anticodon interactions. Each ribosome variant system was minimized with relaxed restraints using steepest descent and conjugate gradient PR, and equilibrated in solvent with physiological salt conditions, as described in the literature [45,46,47,48,49]. After equilibration, each system was allowed to run an MdMD for a biased simulation length of 50 nanoseconds (of MdMD time), which samples over time faster than standard MD. The protocol for refinement included the following steps: (1) Minimization with explicit water molecules and ions; (2) energy minimization of the entire system; and (3) MDS for >10 ns to relax to the force field (AMBER03). Following the refinement protocol, production MdMD simulations of >50 ns were completed to collect data.

AMBER03 force fields were used with the current release of NAnoscale Molecular Dynamics 2 engine [50,51]. The ribosomes systems simulated, including hydrogens, consist of 2.4–2.5 × 10^5^ atoms prior to solvation with TIP3P water and ions. In all cases, we neutralized with counter-ions, maintained crystallographically determined Mg2+ ions with coordinated waters at phosphate backbone positions, and then created a solvent with 150 mM Na^+^ Cl^−^ to recreate physiological strength. TIP3P water molecules were added around the protein at a depth of 15–18 Å from the edge of the molecule depending upon the side [52]. Our protocol has been previously described in the literature [48]. Solvated protein simulations consist of a box with 2.9–3.1 × 10^6^ atoms including nucleic acids, proteins, counter-ions, solvent ions, and solvent waters.

Simulations were carried out using the particle mesh Ewald technique with repeating boundary conditions with a 9 Å nonbonded cut-off, using SHAKE with a 2-fs timestep. Pre-equilibration was started with three stages of minimization with 100,000 steps of SD PRCG, gradually relaxing restraints, followed by 4 ns of heating under MD, with the atomic positions of nucleic and protein fixed. Then, two cycles of minimization (50,000 steps each) and heating (2–10 ns) were carried out with soft restraints of 10 and 5 kcal/(mol·Å^2^) applied to all nucleic and protein backbone atoms. Next, 50,000 steps of minimization were performed with solute restraints reduced to 1 kcal/(mol·Å^2^). Following that, 40 ns of MDS were completed using relaxing restraints (1 kcal/(mol·Å^2^)) until all atoms were unrestrained, while the system was slowly heated from 1 to 310 K using velocity rescaling until reaching the desired 310 K during this equilibration phase. Additionally, NPT equilibration-based MD was used with velocity rescaling for 10–50 ns during equilibration.

Finally, production runs of MdMD were carried out with constant pressure boundary conditions (relaxation time of 1 ps). A constant temperature of 310 K was maintained using the Berendsen weak-coupling algorithm with a time constant of 1 ps. SHAKE constraints were applied to all hydrogens to eliminate X-H vibrations, which allowed for a longer simulation time step (2 fs). Our methods for equilibration and production run protocols are in the literature [45,46,53,54]. Equilibration was determined from a global flattening of RMSD over time after an interval of ~10 ns or similarly with total energy (during equilibration phase). Translational and rotational center-of-mass motions were initially removed. Periodically, simulations were interrupted to have the center-of-mass removed again by a subtraction of velocities to suppress the “flying ice-cube” effect [55]. Following the simulation, the individual frames were superposed back to the origin, to remove rotation and translation effects. Archived snapshots were recorded every successful MdMD sprint, which has an MdMD time stamp of 2 ps, yielding >25,000 snapshots per simulation.

While long unbiased simulations and shorter enhanced sampling simulations are not precisely identical, there is theoretical precedent for the usage of enhanced sampling methods with ribosomes and nucleic acids [56]. In order to adequately sample conformational space to accomplish our specific research goal, we used the Maxwell’s demon molecular dynamics (MdMD) algorithm for our simulations [23]. MdMD has been used successfully to model dynamics of the ribosome [45,48], as well as other systems [49,57]. MdMD is an enhanced sampling method that captures the equivalent of microseconds of unbiased MD simulation data in nanoseconds. This is accomplished by running sprints of MD and comparing the outcome of each sprint against one or more global variables, which are derived from the desired end state configuration of the system. Unlike most other biasing algorithms that are enthalpically driven, MdMD is an entropically driven bias. We collect and amalgamate only sprints that progress towards the desired end state of the system and summarize discarded sprint outputs in a log, where we can estimate a relative time conversion between MdMD versus its equivalent amount of sampling with unbiased MD simulation. Here the “demon” biaser was percent change in structural fluctuations of the global structure (as an average) compared to the previous archived conformation, such that the percent change has increase as compared to the previous archived state to better assess quicker changes in the global structure over desired interval. In the cases of our simulations, these MdMD time equivalencies are approximately 1300 ns and relevant calculations are described in the supplementary material. Typical scaling factors in past simulations have ranged between 25 and 100 times over the course of an MdMD simulation [47,48,49].

## 3. Results

The failure of U36C to produce frameshifting was not consistent with the doublet model, motivating our molecular dynamics (MD) and MMB study. MD refers to the calculation of the time-domain motion of atoms in molecules, typically with all atoms mobile and often immersed in solvent. The ribosome is a particularly challenging system to model by MD, due to its very large size and extensive conformational changes occurring over long time scales. MD simulations typically run over time scales of weeks or even months, and iteration is typically needed leading to considerable cost in labor, calendar, and computer time. For these reasons, relatively few research groups pursue MD simulations of the ribosome. Here, we utilized an MdMD biasing approach to enhance conformational sampling, though the cost is still expensive computationally.

However, much of the ribosome’s motion conserves the structure of certain domains, thus it is amenable to a multiscale [58] approach. MacroMoleculeBuilder (MMB) is an internal-coordinate, multiscale modeling code, which has been used for ribosomal morphing [32], homology modeling [59], fitting to low-resolution density maps [60], and other tasks. In particular, it modeled the first all-atom trajectory of the tRNA translocation completion step [32]. Because MMB is so fast, it can be used as a prototype MD tool, to cycle through many iterations as the user explores various what-if scenarios [61]. For example, one can test the feasibility of structural conformations and dynamical trajectories [32], attempt to enforce base pairs or other contacts based on structural data or hypotheses [35], and explore the influence of water, ions, and the physical environment on local or global structure [61]. It is often still useful and/or necessary to confirm findings with MD, but MMB provides a well-behaved, clash free structure and often a clear, focused hypothesis, typically resulting in one or two (rather than many) iterations of the expensive MD step [61].

We used MMB to create models of frameshifted tRNAs following the doublet decoding model. Our final, non-double model was not known to us at that time; therefore, the modeling was not biased by this idea. In our constructed structural model, U36 and U36C were structurally nearly identical, aside from the chemical groups at pyrimidine ring positions 3 and 4 (Figure 3). We also constructed a U36C-G1A model, in which G1A is a substitution at codon position 1. U36C-G1A was constructed after the U36 and U36C MD results but was structurally as close as possible to the latter two models, again with the exception of the tRNA and mRNA mutations, again following the doublet decoding model (Figure 3). This last would be equivalent to CCG-reading tRNA^Gly^ frameshifting at an ACA Thr codon—though no such a phenomenon has been observed experimentally to our knowledge.

**Figure 5:**
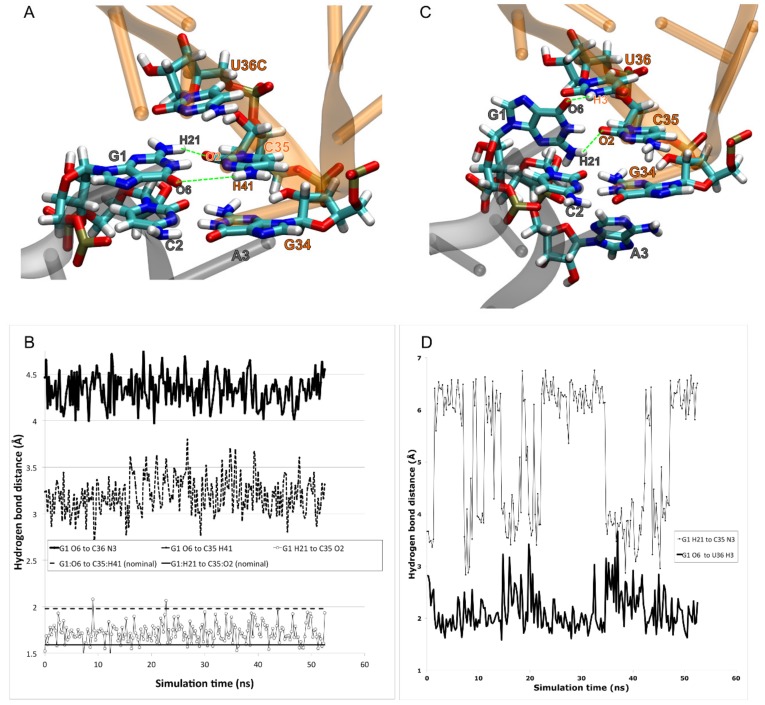
MdMD simulations indicate that the frameshifted configuration of tRNA^Ser3^ at Ala codons is stabilized by an unexpected interaction between codon position 1 (G1) and anticodon position 1 (U36), rather than by doublet decoding, explaining the loss of binding in the U36C mutant. (**A**) We prepared the tRNA^Ser3^ U36C mutant in the doublet decoding configuration. The two codon–anticodon base pairs (G1-C35, C2-G34) were approximately maintained during minimization and simulation. (**B**) The hydrogen bonds, however, were longer than has been observed elsewhere, suggesting that this interaction is not strong. (**C**) We similarly prepared the wild type (U36) in the doublet configuration, but G1 rotated away from C35 to interact instead with U36. (**D**) The hydrogen bond between G1:O6 and U36:H3 appeared strong and persistent. A weak interaction occurred between G1:H21 C35:N3.

The U36 and U36C MDS’ were produced first. The U36 simulation yielded the key surprising result during the minimization and equilibration stage (prior to the production MD run): U36 remained close to its original modeled position, but G1 had flipped to create a strong and persistent hydrogen bond between codon G1:O6 and tRNA U36:H3 (Figure 5). This was evidently favorable in this simulation system, compared to a C35-G1 Watson–Crick base pair. Why would a C35-G1 not be favorable? The answer was given by our U36C simulation, which of course did not have a U36C:H3 hydrogen-bond donor.

**Figure 6:**
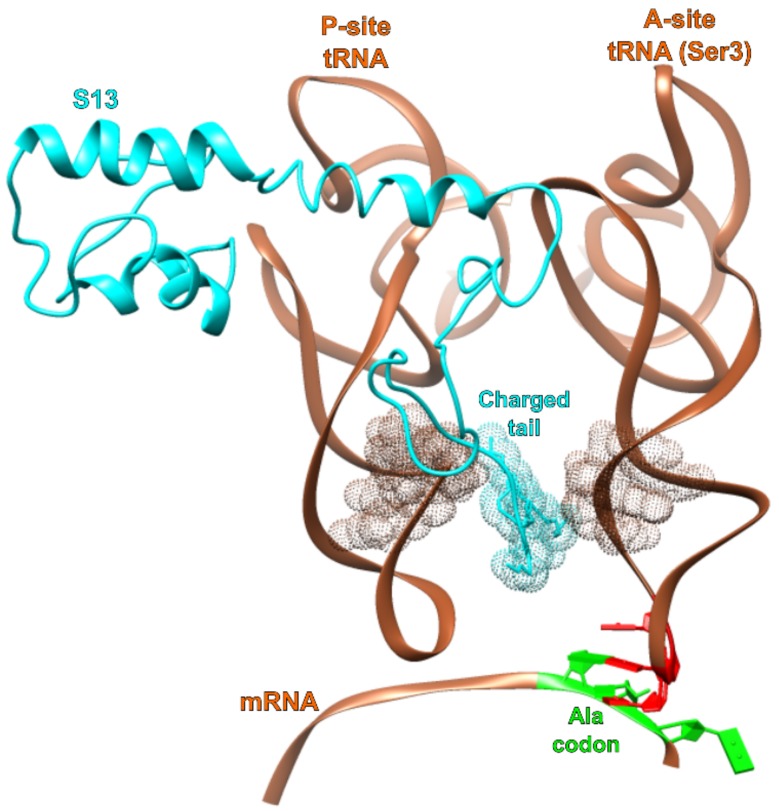
Interaction of S13 with tRNA A and P site tRNAs. The C-terminal region of ribosomal protein S13 (teal) inserts between the A- and P-site tRNA (copper) anticodon stems. This reduces the intervening space and may produce entropic pressure against adopting the −1 frameshifting conformation.

In the U36C simulation, the C35-G1 Watson–Crick interaction in the MMB model was maintained throughout the simulation, following the doublet decoding model. However the hydrogen-bond lengths were significantly longer than in the crystallographically observed Watson–Crick interactions [34]. Therefore, the base pairing interaction appears highly strained (Figure 5). The reason for the strain is not immediately obvious, as there is plenty of distance between the A and P site tRNA anticodon stems. The C-terminus of protein S13, however, is inserted between the tRNAs. The terminus of S13 is known play a role in suppressing frameshifting [62]. Our results suggest a mechanism for this; possibly the S13 C-terminus is an entropic hindrance to the −1 frameshifted conformation. This is illustrated in Figure 6.

We then hypothesized that a double mutant, U36C-G1A, would give rise to a U36C:N3 G1A:H61 hydrogen bond, similar to the G1:O6 to U36:H3 hydrogen bond. However, this did not occur in the MDS; instead, G1A remained in an interaction with C35, as in the MMB model. (Figure 3) It could be that the geometries are different enough that this hydrogen bond may not be as favorable as the G1:O6 to U36:H3 one.

## 4. Discussion

How does tRNA^Ser3^ promote −1 frameshifting on the GCA codon? Our results suggest that these tRNAs, in the context of the ribosomal A site, can adopt non-standard codon–anticodon interactions. We initially hypothesized that this could involve a non-standard anticodon loop 1:6 stacking (one on the 5′ side and six on the 3′ side), instead of the standard anticodon loop 2:5 stacking that would subsequently flip back to the standard stacking at the P-site after translocation [63], following the doublet model. Models of ASL^Ser^_GCU_ with 1:6 stacking, making two base pairs with a GCA codon in standard mRNA conformation in the ribosomal A site, showed that that this is sterically feasible; this model was then the input to an MD simulation. It was during MD simulations (already at the minimization stage) that we observed a switch to a non-doublet conformation. Thus, our initial modeling (Figure 4) did not unduly bias the simulation, rather the unusual interaction appears strongly driven by the underlying physics.

The unusual conformation predicted by the MD follows. mRNA position 2 is still interacting with tRNA residue 34, just as in the doublet decoding model. However, mRNA position 1 is *not* interacting in a Watson–Crick base pair with tRNA residue 35. Rather, the position 1 residue makes a persistent hydrogen bond with residue U36, and a weaker and less consistent hydrogen bond with residue C35, straddling the two anticodon residues. The hydrogen bond with U36:H3 cannot occur in U36C because cytosine has no H3. We ran a simulation with U36C which confirms that the U36C-G1 interaction does not occur. This could explain kinetics experiments performed by a collaborator, which showed the U36C suppressed frameshifting (currently unpublished data).

The U36C simulation also allowed us to probe a hypothetical G1-C35 Watson–Crick base pairing interaction. The hydrogen bonds of this base pair appear longer than in the canonical [34] base pair (Figure 2), indicating strain, as if the −1 frameshifting were crowding the A and P site tRNAs. There is a substantial gap between the two tRNAs, but the gap is occupied by the tail of protein S13. Since truncating this tail is known to increase −1 frameshifting, we speculate that one of its roles is to maintain a minimum physical distance between A and P site tRNAs, as part of a frameshifting prevention mechanism. Without this tail, one can imagine that observed strain would disappear, lowering the energy associated with the doublet decoding configuration. This would, in turn, explain the increased frameshifting rate.

## 5. Conclusions

How does tRNA^Ser3^ induce frameshifting at alanine codons? For over three decades, the explanation has been doublet decoding, based on Bruce et al.’s observation that tRNA (anticodon) bases 35 and 34 are complementary to bases 1 and 2 of the codon. For most of that time we have known that the isosteric U36C mutation does not enter the model, but does eliminate frameshifting. An unspecified conformational rearrangement in the anticodon was postulated to explain this. The frameshifted conformation resisted structural biology (possibly due to stability issues) and so for lack of a better hypothesis, doublet decoding persisted. The “computational microscope” steps in when working with rare events and unstable structures, and in this case offered an explanation as remarkable as it is simple—a rotation of base 1 in the codon, trading a strained attempt at a C35-G1 Watson–Crick base pair for a single but strong hydrogen bond with U36. Why was the C35-G1 base pair strained? Examining the simulation trajectory (or even the experimental structures) provided a possible culprit—the C-terminal tail of S13, inserted between the tRNA^Ser3^ and its upstream neighbor, facing tighter quarters now that tRNA^Ser3^ was shifted upstream by one mRNA position. This, in turn, would explain Cukras and Green’s observation that trimming the tail increases frameshifting.

## Supplementary Materials

The following are available online at https://www.mdpi.com/2218-273X/9/11/745/s1, Figure S1: title, Table S1: title, Video S1: title. Calculations for conversion between MdMD biased and unbiased simulation time: Sprint_avg_(N_avg_) = T_unbiased_, where Sprint is 2ps or 10 ps and N_avg_ is average number of attempts to get to next archived sprint run (typically 10–15 times on average), which results in effective time sampling of 25 ps per archived frame of MdMD. Example: U36: MD_sprint-size_ × N_rejection-rate_ = (2ps × 10–15) = ~25 ps per sprint.
