# Supplementary material for: Molecular Dynamics Simulations Suggest a Non-Doublet Decoding Model of −1 Frameshifting by tRNASer3
Source: Biomolecules. 2019 Nov 18;9(11):745. doi: 10.3390/biom9110745 (PMC6920855; doi:10.3390/biom9110745)
Supplement: Supplementary file 1 [file biomolecules-09-00745-s001.pdf]

SUPPLEMENTARY INFORMATION

Molecular Dynamics Simulations Suggest a Non-Doublet Decoding Model of –1 Frameshifting by $tRNA^{Ser3}$

Thomas Caulfield [1,*], Matt Coban [2], Alex Tek [3] and Samuel Coulbourn Flores [4,*]

[1] Department of Neuroscience, Mayo Clinic, Jacksonville, FL 32224, USA
[2] Department of Cancer Biology, Mayo Clinic, Jacksonville, FL 32224, USA
[3] Department of Cell and Molecular Biology, Uppsala University, 751 24 Uppsala, Sweden
[4] Department of Biochemistry and Biophysics, Stockholm University, 10691 Stockholm, Sweden
***** Correspondence: TC: caulfield.thomas@mayo.edu (T.C.); samuel.flores@dbb.su.se (S.F.)

Received: 29 October 2019; Accepted: 14 November 2019; Published: 18 November 2019

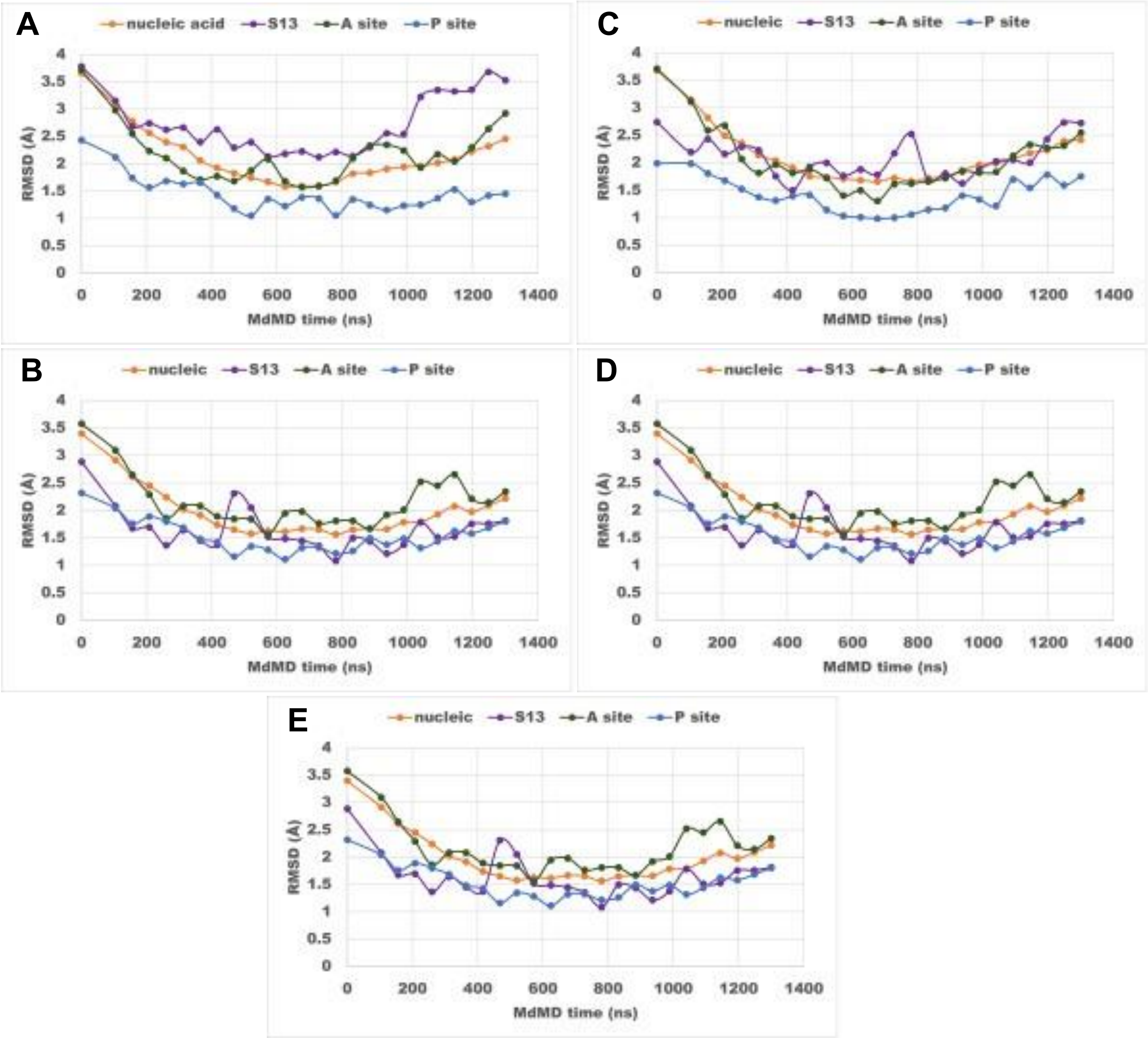

Figure S1: Various average RMSD measurements over the course of MdMD simulations. To generate these data, structures and trajectories were loaded into VMD (latest version) and analyzed. The translation and rotation were removed by aligning the structures to the first frame. The color selection and calculated alignments, which results in the subsequent average RMSD measurement over the course of the MdMD simulations are as follows: nucleic acid (orange), the entire nucleic acid content of the system; S13 (purple), only the protein S13; A site (green), only the A site nucleic acid; P site (blue), only the P site nucleic acid. **A**, the system with an alanine anticodon and its cognate mRNA codon. **B**, the system with the aforementioned U33 “grapple” structure. **C**, the system with WT tRNAser (U36) in the doublet decoding configuration, but with G1 rotated to hydrogen bond to U36:H3 in a stacked arrangement. **D**, the system with U36C mutated tRNAser in the doublet decoding configuration, with base pairing between G1-C35 and C2-G34. **E**, the system with U36-A1913 interaction.

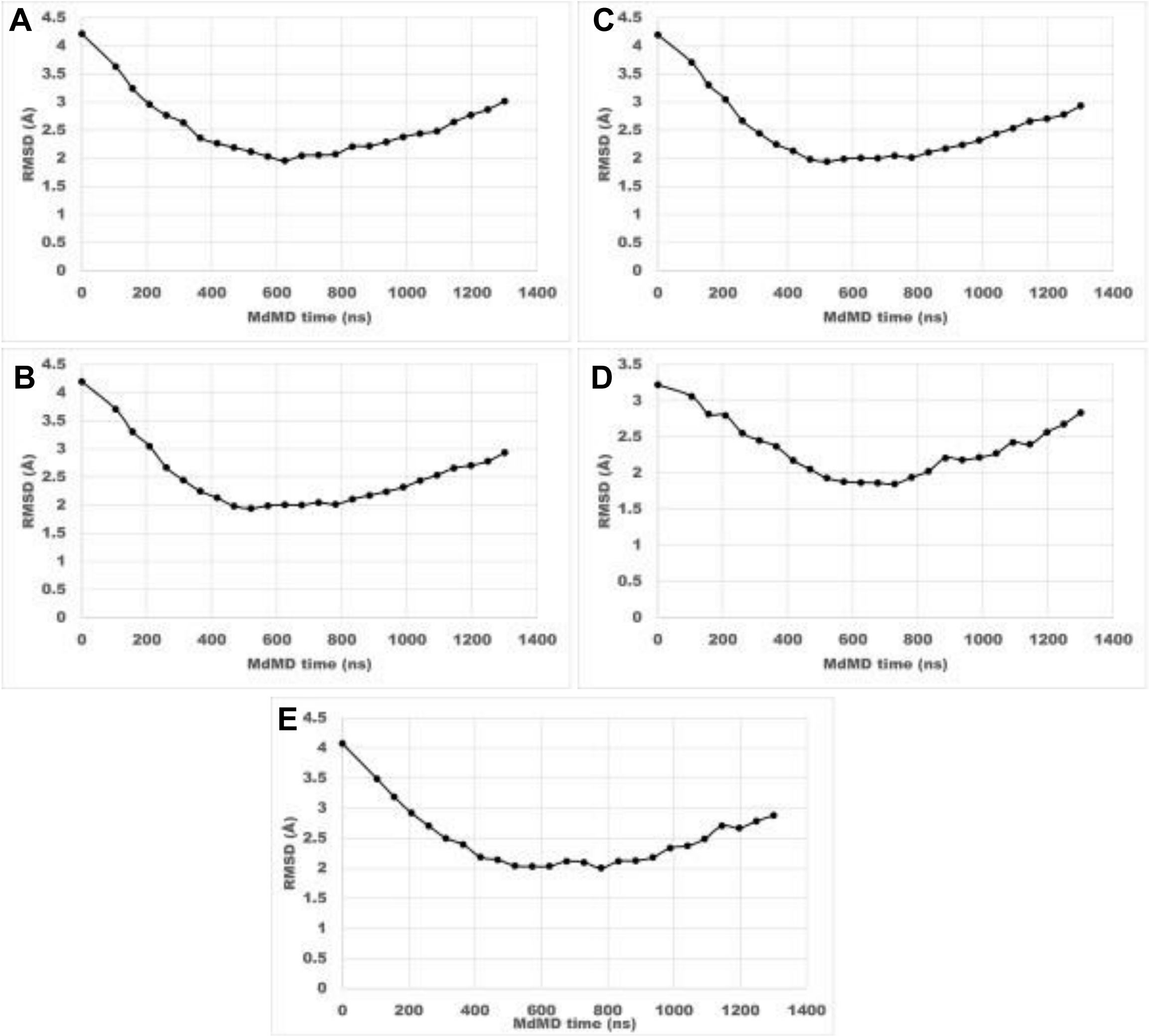

Figure S2: Average RMSD measurements for all ribosomal protein over the course of MdMD simulations. To generate these data, structures and trajectories were loaded into VMD (latest version). The translation and rotation were removed by aligning the structures to the first frame. All protein in the system was the selection for alignment and subsequent average RMSD measurement over the course of the MdMD simulations. **A**, the system with an alanine anticodon and its cognate mRNA codon. **B**, the system with the aforementioned U33 “grapple” structure. **C**, the system with WT tRNAser (U36) in the doublet decoding configuration, but with G1 rotated to hydrogen bond to U36:H3 in a stacked arrangement. **D**, the system with U36C mutated tRNAser in the doublet decoding configuration, with base pairing between G1-C35 and C2-G34. **E**, the system with U36-A1913 interaction.

<u>Supplemental Structure Files:</u>
Multiframe PDBs are snapshots representing each system at 25 timepoints of the MdMD simulation. Due to the enhanced sampling of the MdMD algorithm, as described in the main text, each of these timepoints is approximately equivalent to 52ns of unbiased simulation, about 1300ns overall.

Each of the 5 PDBs describes a different system, more detailed descriptions of the systems and relevant interactions is included in the text. To summarize, the full system includes ribosomal proteins, particularly S13 (chain M), the A (chain Y), P (chain V), and E (chain W) sites, 30S and 50S ribosomal subunits (chain A).

Files are given as:

1) ALAamber__ribosomeSimMMD_2014_25snapshots.pdb contains the tRNAala and its cognate codon.
2) U33grappleUW__ribosomeSimMMD_2014_25snapshots.pdb contains the U33 grapple structure base pairing with mRNA A3.
3) U36stacked_renum__ribosomeSimMMD_2014_25snapshots.pdb contains the WT tRNAser in a doublet decoding configuration, except with an mRNA G1 to U36:H3 hydrogen bond.
4) U33grappleUW__ribosomeSimMMD_2014_25snapshots.pdb contains the U36C substituted tRNAser in a doublet decoding configuration
5) YU36_aA1913__ribosomeSimMMD_2014_25snapshots.pdb contains the WT tRNAser, but with an interaction formed between tRNA U36 and 23S A1913
